# Effects of tiered cardiac rehabilitation on CRP, TNF-α, and physical endurance in older adults with coronary heart disease

**DOI:** 10.1515/biol-2022-1040

**Published:** 2025-05-20

**Authors:** Cong Luo, Lan Li, Lan Hou, Fengjiao Shi

**Affiliations:** Department of Cardiovascular Medicine, The Fourth Hospital of Changsha, Changsha, 410219, China; Ward of Neurology, The Fourth Hospital of Changsha, Changsha, 410006, China

**Keywords:** stratified cardiac rehabilitation, elderly, coronary heart disease, CRP, exercise tolerance

## Abstract

Coronary heart disease (CHD) is a highly prevalent disease in the elderly population, with atherosclerosis as its pathology, which can also be viewed as a chronic inflammatory response of the organism. Regular moderate-intensity exercise can direct the immune response toward an anti-inflammatory state, which is beneficial for improving the health and exercise tolerance. In cardiac rehabilitation, attention to the management of inflammatory factors as well as the improvement of exercise endurance is beneficial for the rehabilitation of elderly patients with coronary artery disease. This study investigates the impact of tiered cardiac rehabilitation programs on levels of C-reactive protein (CRP), tumor necrosis factor-alpha (TNF-α), and the capacity for physical exertion in older CHD patients. From March 2020 to April 2022, 94 elderly patients with CHD visiting our institution were recruited and randomly allocated into either a control group or an observation group, each comprising 47 participants. The standard care group participated in traditional rehabilitation exercises, whereas the experimental group received customized, tiered, cardiac rehabilitation interventions. We assessed the variations in CRP and tumor necrosis factor alpha (TNF-α) levels, along with exercise capacity, before and after treatment in both groups. The result shows that significant reductions in CRP and TNF-α levels were seen in the experimental group after 4 and 12 weeks, compared to the standard care group. Analysis showed clear trends in CRP and TNF-α changes over the interventions, with the experimental group showing better results. CRP levels decreased consistently, while TNF-α levels stayed stable. The experimental group also showed improvements in physical endurance measures compared to the control group. Interleukin 6 (IL-6) and fibrinogen (Fib) in the observation group decreased compared with the control group (*P* < 0.01). After 12 weeks of treatment, CRP and TNF-α showed significant negative correlation with exercise endurance index – 6 min walking test (6 MWT), anaerobic threshold (AT), maximum oxygen consumption (VO_2max_), and exercise duration (ED); significant positive correlation between cardiac rehabilitation grade and exercise endurance index (6 MWT, AT, VO_2max_ and ED); and both groups experienced cardiovascular adverse events and exercise muscle injury. The analysis shows that the graded nursing of cardiac rehabilitation can effectively reduce the levels of CRP, TNF-α, IL-6, and Fib in elderly patients with CHD and significantly improve the exercise endurance of patients with good safety.

## Graphical abstract

**Figure:**
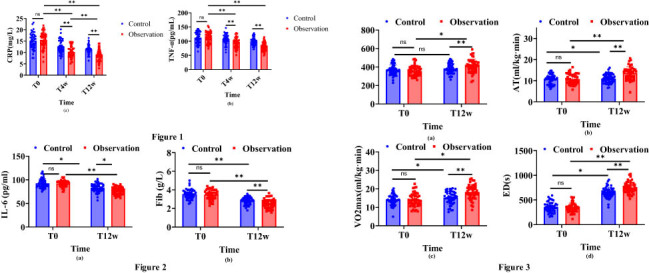

## 1 Introduction

With the evolving burden of coronary heart disease (CHD) and related risk factors worldwide, CHD has become a serious challenge to the health of elderly populations [1]. In the last three decades, the age-standardized prevalence of CHD has seen a 4.6% reduction, while absolute deaths have decreased by 31% and disability-adjusted life years (DALYs) have dropped by 28.6% [2]. These data underscore significant advancements in both the prevention and management of CHD worldwide. Nevertheless, it is vital to address the health issues that elderly CHD patients face, which are exacerbated by lifestyle shifts and an aging population [3]. A recent prospective chronic disease study in China links increased physical activity with lower cardiovascular mortality, suggesting that enhanced physical activity could mitigate the death risk among cardiovascular disease patients [4]. Thus, boosting physical activity might be a viable approach to enhance the health of elderly CHD patients.

C-reactive protein (CRP), an acute phase protein, tends to rise during inflammation or tissue damage. For those suffering from CHD, a rise in CRP levels often indicates an inflammatory reaction to atherosclerosis. Higher levels of CRP are linked to a greater risk of cardiovascular incidents and correlate closely with their severity [5,6]. Similarly, tumor necrosis factor-α (TNF-α), a regulator of inflammation and immune responses, when overproduced in CHD patients, can heighten the inflammation and expedite atherosclerosis progression [7]. Elevated TNF-α levels are tied to heightened risks of cardiovascular events and myocardial damage [8]. Furthermore, a decline in exercise tolerance characterizes many elderly CHD sufferers, severely impacting their quality of life (QoL). This decline is associated with measures of vascular wall stiffness [9,10]. Monitoring CRP and TNF-α levels along with exercise tolerance shifts is crucial for the effective diagnosis and management of CHD in older adults.

At present, intensity interval training and resistance exercises are the main rehabilitation training methods for patients undergoing cardiac rehabilitation. However, this training is not suitable for the elderly population [11]. High-intensity interval training is recognized as an effective modality for improving cardiorespiratory fitness (peak oxygen consumption [VO_2_]) in patients with coronary artery disease (CAD) [12]. Exercise-based cardiac rehabilitation has been shown to improve HRQoL and hospital admissions in patients with heart failure and may reduce mortality in the long term, and these reductions appear to be consistent across patient and program characteristics [13]. Home-based cardiac rehabilitation had the best results in terms of functional capacity, QoL, and reduced readmission rates within 90 days [14]. It is clear that scientifically based cardiac rehabilitation is effective in improving cardiorespiratory fitness and reducing the risk of readmission in patients with CHD, but there is a lack of targeted research on exercise tolerance and inflammatory factors. Stratified cardiac rehabilitation was first proposed by Patti et al. [15] as a dynamically adjustable rehabilitation method for patients with cardiovascular diseases [16]. However, reports on its effects on inflammatory markers and exercise tolerance in elderly CHD patients are still lacking. Thus, this study aims to provide new evidence for improving the prognosis of these patients by observing the impact of stratified cardiac rehabilitation on CRP, TNF-α, and exercise tolerance.

## 2 Data and methods

### 2.1 General data

Sample size was calculated using MACE medical software, using the superiority of the two independent samples, with the CRP level, the mean of 80 patients was 10.5 within 1 year, the mean of 8.5, 8.5, the combined standard deviation of 3.6, clinical significance difference between the two groups, 0.05, *α* 0, power = 1 − *β* = 0.9, a sample size ratio of 1:1, considering a 10% loss-to-visit rate, and the final sample size was 47. Ninety-four senior patients diagnosed with CHD were enlisted and categorized into a control group and an observation group, each comprising 47 individuals, utilizing a random number table selection method. The inclusion criteria were as follows: (1) diagnosis of CHD via coronary angiography or CT angiography; (2) aged 60 years or older; (3) agreement to participate in the study through signed informed consent. The exclusion criteria included the following: (1) history of severe cardiovascular incidents like acute myocardial infarction or heart failure; (2) presence of serious liver or kidney dysfunction, malignant cancers, or other major systemic conditions; (3) significant cognitive disabilities that impair treatment cooperation; (4) recent (within 3 months) use of medications that could skew the study results; (5) poor adherence to medical advice impacting treatment; (6) other systemic diseases that could influence the survival. No significant statistical differences were observed between the groups in terms of gender, age, duration of illness, NYHY cardiac function classification, comorbidities, and medication usage (*P* > 0.05), indicating a balanced baseline data set (Table 1).

**Table 1:** Comparison of general data for the two patient groups

Group	Number of cases	Male/Female	Age (years)	Course of disease (year)	NYHY functional class				Comorbidities			Type of medication (Case)				
					I	II	III	IV	Hypertension [cases (%)]	Dyslipidemia [cases (%)]	Type 2 diabetes mellitus [cases (%)]	Lipid-lowering drugs	β-blockers	ACE inhibitor	Other drugs	Co-administered drugs
Control group	47	28/19	65.6 ± 5.2	5.1 ± 2.3	12	20	9	6	32 (68.1)	15 (31.9)	11 (23.4)	36	34	31	12	37
Observation group	47	27/20	64.9 ± 5.6	4.8 ± 2.1	13	19	10	5	30 (63.8)	14 (29.8)	10 (21.3)	34	36	33	10	40
*χ* ^*2*^ */t*		0.044	−0.682	0.243	0.209				0.190	0.050	0.061	0.224	0.224	0.196	0.237	0.646
*P*		0.834	0.497	0.809	0.976				0.663	0.823	0.804	0.636	0.636	0.658	0.626	0.421


**Informed consent:** Informed consent was obtained from all individuals included in this study.
**Ethical approval:** The research related to human use complied with all the relevant national regulations, institutional policies, and in accordance with the tenets of the Helsinki Declaration and was approved by the Ethics Committee of The Fourth Hospital of Changsha.

### 2.2 Intervention methods

All patients received conventional drug therapy. For example, aspirin and clopidogrel can be used to guide patients to eat a reasonable diet and control weight.

The control group underwent standard rehabilitation therapy, which involved a structured program of power cycling, segmented into warm-up, cycling, and cooling down phases, followed by walking exercises. Specifically, the regimen included a 5-min warm-up, 20 min of cycling, and a 5-min cool-down, culminating in 30 min of total exercise time. Walking exercises were conducted 1-h post-cycling, with each session lasting for 20 min, and the protocol was set for 5 days per week. The schedule was adapted based on individual patient recovery progress. Over the 12-week program, the care team diligently monitored vital signs during sessions, and key cardiovascular metrics such as blood pressure, heart rate, and ECG were routinely assessed weekly to timely identify any progression in heart conditions.

In the observation group, stratified cardiac rehabilitation was implemented on top of the control group. Initially, a stratified cardiac rehabilitation plan was developed, and multiple batches of cardiology head physicians, supervising nurses, and cardiac rehabilitation nurses were trained. The attending physician undertook the patient grading assessment, the attending nurse was responsible for screening suitable patients, and the cardiac rehabilitation nurse was responsible for assisting patients in establishing rehabilitation goals and carrying out early activity and pulmonary function rehabilitation intervention. Stratified cardiac rehabilitation was divided into seven levels. Each level was divided into two aspects: early activity program and breathing training program. Specific contents: (1) Level 1: the bleeding risk of the patient was assessed by the attending physician after admission. Passive exercises in bed, active/passive joint activities in a semi-recumbent position, or active/passive reactive bicycle in a recumbent position was performed for patients without bleeding risk for 20–30 min once a day. At 10 o’clock every day, respiratory control exercises or recumbent breathing exercises were performed for 20 min once a day for 1 week. (2) Level 2: When the level I was completed and can be carried out stably for 7 days, assessment was done to find out whether the hemodynamic indexes of patients were stable. The stabilized patients received sitting exercises and sitting breathing exercises, while the unstabilized patients continued with level 1 training and reassessed after an extension of 3 days. Sitting exercises and sitting breathing exercises, bedside sitting training, active joint activities, or sitting active/passive reactive treadmill in bed were done 1 h/day. Sitting breathing exercises involved performing abdominal respiration in the sitting relaxed state, inhalation through the nose and exhalation through the mouth for 5–10 min, for 1 week. (3) Level 3: The patients were asked to stand for 2 min after the second level. If the patient can start the third level training for 2 min, if the independent standing time was less than 2 min, the second level training was evaluated again after 3 days, the main intervention method was 1 h/day, divided into active joint activity, active power riding, lower limb strength exercises for 1 week; and standing breathing exercises were done for 5–10 min for 1 week. (4) Level 4: After the third stage was completed, 50 m walking test and evaluation were carried out. If patients can normally complete the 50 m walking test, they will start level 4 training; if the distance of 50 m walking is less than 50 m, they will continue level 3 training and reassessed after 3 days, and so on. Level 4 intervention was mainly based on incremental walking training and sitting Eight Pieces of Brocade (Ba Duan Jin). During each training, the time and distance of walking should be gradually increased, which can be gradually increased to 20–30 min/day according to individual conditions, 3–5 times/week. The sitting Eight Pieces of Brocade (Ba Duan Jin) was performed 30 min after the incremental walking training for 2 weeks. (5) Level 5: After the fourth level was completed, five-level training will be carried out. If the 200 m walking distance is less than 150 m, the fourth level of training will be continued and evaluated again after 3 days. The rest can be done in the same manner. For the fifth level, 200 m walking training and standing breathing exercises were used as the main intervention methods. During each training, the walking time and distance should be gradually increased, which can be gradually increased to 20–30 min/day and 2–3 times/day according to individual conditions, and the training included active joint movement and lower limb strength exercises. The standing breathing exercises were performed 30 min after the 200 m walking training for 2 weeks. (6) Level 6: After the fifth level was completed, a 500 m walking test and evaluation shall be carried out. If the patient can normally complete the 200 m walking test, he/she will start the fifth-level training; if the distance is shorter than 200 m, he/she will continue the fifth-level training, and re-evaluate after 3 days, and so on. The sixth level was mainly intervened by 500 m walking training and Taijiquan. During each training, the patient can move freely in the ward area, and the walking time and distance were gradually increased. The intervention time can be gradually increased to 20–30 min/day, 3–5 times/week according to individual conditions. Taijiquan was performed 30 min after incremental walking training for 2 weeks, and the corresponding reexamination plan was made. (7) Level 7: After the completion of level 6, a 500 m walking test and evaluation shall be carried out again. If the patient can normally complete the 500 m (≥370 m) walking test, level 4 training shall be started; if the 50 m walking distance is less than 370 m, level 6 training shall be continued, and the assessment shall be carried out again after 3 days, and so on. Level 7 intervention methods include independent activity in the ward and standing Eight Pieces of Brocade (Ba Duan Jin). During each training, the range of activity and activity time shall be gradually increased. The intervention time can be gradually increased to 20–30 min/time, 2 times/day, and 3–5 times/week according to individual conditions. The standing Eight Pieces of Brocade (Ba Duan Jin) was carried out 30 min after spontaneous activity in the ward and continued until 12 weeks of intervention (see Table 2 for details).

**Table 2:** Stratified cardiac rehabilitation plan

Level	Objective	Assessment	Assessment result	Method
Level 1	Prevent complications	Assess if there is a risk of bleeding	Rehabilitation training was initiated without any risk of bleeding	Early activity: passive movement in the bed, 20–30 min, 1/day for 1 week
	Enhance exercise tolerance			Respiratory training: adjust breathing, 20 min, 1/day for 1 week
Level 2	Enhance exercise tolerance	Check for hemodynamic stability	Rehabilitation training was initiated without any risk of bleeding	Early activities: sitting practice, 1 h/day for 1 week
	Improve cardiopulmonary function			Breathing training: sitting breathing exercise, lasting for 5–10 min, lasting for 1 week
Level 3	Increase activity distance	Is there any problem when standing for 2 min?	Rehabilitation training was initiated without any risk of bleeding	Early activities: increased distance walking and training
	Provide dietary guidance upon discharge			Respiratory training: 20–30 min/day, 3–5 times/week for 2 weeks
Level 4	Increase walking distance and provide post-discharge activity instructions	Can pass the 50 m walking test?	Effectively complete	Early activities: increased distance walking and training
				Respiratory training: 20–30 min/day, 3–5 times/week for 2 weeks
Level 5	Reinforce post-discharge activity instructions.	Can pass the 200 m walking test?	Effectively complete	Early activities: 200 m to walk 2–3 times/day
				Respiratory training: standing breathing exercises for 20–30 min/day, 2–3 times/day
Level 6	No angina after activity	Can you pass the 500 m walking test?	Effectively complete	Early activities: 500 m to walk for 20–30 min/day, 3–5 times/week, lasting for 2 weeks
	Patient can repeat discharge instructions			Respiratory training: Tai Chi for 30 min, 1 time/day, lasting for 2 weeks
Level 7	Discharge smoothly	Can you complete the 500 m walking test?	Effectively complete	Early activities: the ward was 20–30 min/time, 2 times/day, 3–5 times/week
				Respiratory training: last until 12 weeks of intervention

### 2.3 Observational indicators

#### 2.3.1 General data investigation

Upon admission, each patient was promptly evaluated, gathering basic details such as age, gender, and duration of illness, along with a comprehensive record of their CHD and any associated underlying conditions.

#### 2.3.2 Serological index examination

About 3–5 mL fasting venous whole blood samples were collected before intervention and at 4 and 12 weeks after treatment, centrifuged at 1,000 rpm for 10 min after coagulation, and serum samples were separated for testing. Reagents and instruments: CRP test kit and automatic biochemical analyzer. Immunoturbidimetry was used for CRP. The serum sample was diluted and mixed with a reagent complex, incubated at 37°C for 10 min, followed by measuring the absorbance at a 540 nm wavelength. The CRP levels in the sample were then calculated using a standard curve. For TNF-α measurement, the interleukin 6 (IL-6) and fibrinogen (Fib) double antibody sandwich ELISA technique was utilized. First, the microplate was coated with anti-TNF-α antibody, then the sample and enzyme-labeled detection antibody were added, the substrate was added after washing, and the absorbance value was measured by a microplate reader after the enzymatic reaction. Reagents and instruments: TNF-α, IL-6, Fib ELISA kit, and microplate reader. Operation: Operation was done according to the kit specification, including incubation, washing, color development, termination of reaction, reading of the absorbance value at 450 nm, and calculation of TNF-α concentration. Quality control: Quality control parameters were set up and daily quality control was carried out to ensure the accuracy and reliability of test results.

#### 2.3.3 Exercise tolerance examination

The exercise tolerance of patients was evaluated by a 6-min walking test (6MWT) before intervention and at 12 weeks after intervention. Patients participated in a walking test along a 50 m corridor, with their total walking distance being noted. Concurrently, exercise tolerance metrics, including anaerobic threshold (AT), maximum oxygen consumption (VO_2max_), and exercise duration (ED), were measured using the Smax58ca cardiopulmonary exercise function tester from Nanjing Hanya Health Technology Co., Ltd., both prior to and after 3 months of treatment. Musculoskeletal injuries and cardiovascular adverse events during treatment were also recorded.

### 2.4 Statistical methods

Statistical analysis in this investigation was conducted using SPSS 22.0 software. The data were presented as mean ± standard deviation and subjected to a normality test. An independent sample *t*-test was employed for inter-group comparisons, while a paired *t*-test was used for within-group comparisons. Enumeration data were quantified by case count, with a *χ* ^2^ test applied for between-group analysis. Data from three time points within the group were analyzed by two-way ANOVA to analyze the interaction of graded care for cardiac rehabilitation and usual care on CRP, TNF- α, and exercise endurance, and the post-act test and snk-Q test were done for multiple comparisons. Data from two time points within groups were analyzed by the paired sample *t* test, and *P* < 0.05 was set to be statistically significant. Pearson correlation analysis was carried out to determine the correlation between the data.

## 3 Results

### 3.1 Variations in CRP levels across the two groups

**Figure 1:**
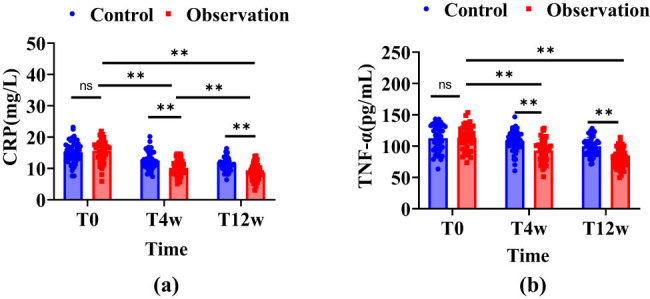
Changes in CRP and TNF-α levels for the two patient groups. Note: In the figure blue indicates the control group, red indicates the observation group, ns is not statistically significant, ** indicates *P* < 0.001, T0 indicates before treatment, T4w is at 4 weeks of treatment, and T12w is at 12 weeks of treatment. (a) Change in CRP levels and (b) change in TNF-α levels.

As shown in Figure 1(a), initial CRP levels showed no significant differences between the groups before the intervention (*P* > 0.05). Following interventions at 4 and 12 weeks, both groups experienced reductions in CRP levels, with a more pronounced decline observed in the observation group. The analysis of CRP level changes at these intervals revealed statistically significant discrepancies between the groups (*P* < 0.001). A repeated measures ANOVA indicated a significant difference in the trends of CRP changes during the intervention between the groups (*F*_between groups = 20.421, *P*_between groups < 0.001), demonstrating a time-dependent decrease in CRP levels (*F*_time = 12.649, *P*_time = 0.001). Nevertheless, the interaction effect of time and group differences was not significant (*F*_time * between groups = 2.965, *P*_time * between groups > 0.05). As depicted in Figure 1(b), no notable differences in TNF-α levels were detected between the groups prior to the intervention (*P* > 0.05). However, both groups recorded a decrease in TNF-α levels at 4 and 12 weeks, with the observation group showing a more substantial reduction. Statistically significant differences in TNF-α level changes were noted between the groups at both the 4 and 12-week marks post-intervention (*P* < 0.001). Repeated measures ANOVA revealed a distinct difference in the trends of TNF-α changes during the intervention between the groups (*F*_between groups = 24.612, *P*_between groups < 0.001), but there was no significant time effect on TNF-α level changes (*F*_time = 0.481, *P*_time > 0.05), and the interaction of time with group differences also lacked statistical significance (*F*_time * between groups = 1.773, *P*_time * between groups > 0.05).

### 3.2 Alterations in IL-6 and Fib levels among the groups

**Figure 2:**
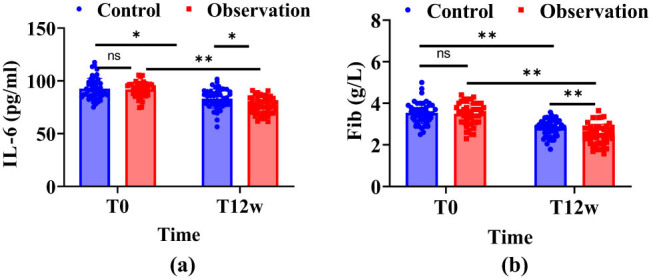
Changes in IL-6 and Fib levels between the two groups. Note: In the figure, blue indicates the control group, red indicates the observation group, ns means not statistically significant, ns indicates *P* > 0.05, * indicates *P* < 0.01, ** indicates *P* < 0.001, T0 is before treatment, and T12w is for 12 weeks of treatment. (a) Changes in IL-6 levels and (b) Changes in Fib levels.

IL-6 and Fib were not compared before treatment (*P* > 0.05), and IL-6 and Fib in the observation group decreased compared with the control group (*P* < 0.01), as shown in Figure 2.

### 3.3 Changes in exercise tolerance between the two groups

**Figure 3:**
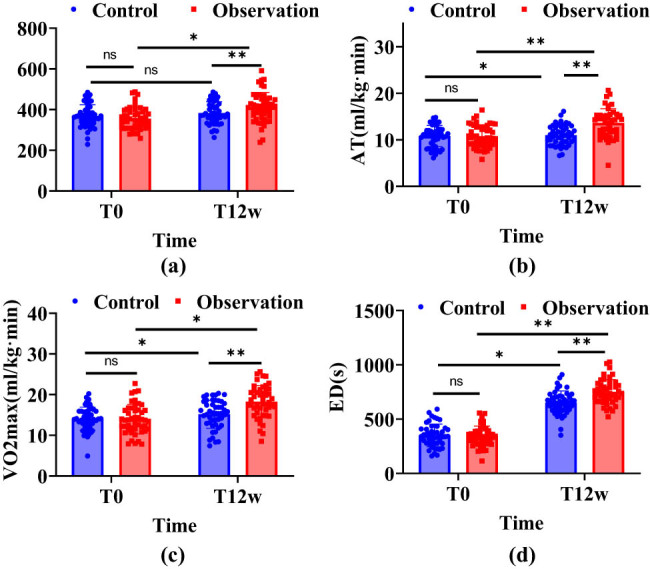
Comparison of changes in indicators related to exercise tolerance between the two groups. Note: In the figure, blue indicates the control group, red indicates the observation group, ns indicates that the comparison between groups is not statistically significant, ns indicates *P* > 0.05, * indicates *P* < 0.01, ** indicates *P <* 0.001, and straight lines and dotted lines indicate the change trend among the three time points of the two groups. (a) 6MWT, (b) AT, (c) VO_2max_, and (d) ED.

Figure 3 reveals that in terms of the 6MWT, no significant enhancement was observed in the control group pre- and post-intervention (*P* > 0.05), while the observation group showed significant improvements (*P* < 0.001). The 6MWT results were notably better in the observation group compared to the control group (*P* < 0.001). Initially, the AT did not differ significantly between the groups (*P* > 0.05). However, post-intervention, both groups saw an increase in AT, with the observation group exhibiting a more pronounced enhancement (*P* < 0.001) and achieving higher AT levels than the control group (*P* < 0.001). Regarding VO_2max_, the control group experienced no notable changes before and after the intervention (*P* > 0.05), in contrast to the observation group, which improved significantly (*P* < 0.001). The VO_2max_ was also higher in the observation group compared to the control group (*P* < 0.001). There were no initial significant differences in ED between the groups (*P* > 0.05). Following intervention, ED increased significantly in both groups (*P* < 0.001), with a more evident extension in the observation group.

### 3.4 Correlation between CRP, TNF-α, and exercise endurance

Before treatment, there was a significant negative correlation between CRP and exercise endurance indicators (6 MWT, AT, VO_2max_, and ED); between TNF-α and exercise endurance indicators (6 MWT, VO_2max_, and ED); between CRP, TNF-α, and exercise endurance indicators (6 MWT, AT, VO_2max_, and ED); between cardiac rehabilitation grade and exercise endurance indicators (6 MWT, AT, VO_2max_, and ED) (Table 3).

**Table 3:** Correlations between CRP, TNF-α, and exercise endurance

Time	Inflammatory factor	6MWT		AT		VO_2max_		ED	
		*r*	*P*	*r*	*P*	*r*	*P*	*r*	*P*
T0	CRP	−0.516	0.000	−0.316	0.002	−0.258	.012	−0.354	0.000
	TNF-α	−0.382	0.000	−0.042	0.687	−0.407	0.000	−0.293	0.004
T12w	CRP	−0.418	0.000	−0.501	0.000	−0.426	0.000	−0.470	0.000
	TNF-α	−0.585	0.000	−0.601	0.000	−0.625	0.000	−0.515	0.000
Stratified cardiac rehabilitation		0.208	0.044	0.451	0.000	0.000	0.000	0.000	0.000

Note: T0 – before treatment; T12w – 12 weeks of treatment.

### 3.5 Safety at the end of the study

There were no musculoskeletal injuries or cardiovascular adverse events in both groups, signaling that the cardiac rehabilitation process had a good safety profile in both groups.

## 4 Discussion

The research indicates a quicker reduction in CRP and TNF-α levels among elderly patients with CHD following stratified cardiac rehabilitation, possibly due to the activation of anti-inflammatory pathways, suppression of inflammatory cell activation, and enhancement of microcirculation [17]. Previous studies link exercise tolerance with muscle functionality and oxygenation levels [18,19]. Stratified cardiac rehabilitation through staged early exercise and respiratory training boosts cardiac output, enhancing tissue oxygenation and promoting vascular regeneration, which in turn augment the aerobic capacity of the patients [20,21]. Elevated levels of CRP and TNF-α can impair cardiomyocytes and myocardial contractility [22]. The suppression of these inflammatory markers through stratified cardiac rehabilitation aids in protecting myocardial cells and overall cardiac function, thus improving exercise tolerance [23]. Enhanced cardiopulmonary function leads to further reduction of inflammatory markers, creating a positive feedback loop in cardiopulmonary health for these patients.

In this study, we observed that cardiac rehabilitation graded care significantly reduced CRP and TNF-α levels in elderly patients with CAD. These findings are not merely observational; they actually reflect the effects of cardiac rehabilitation on inflammatory markers at the cellular and molecular levels. Cardiac rehabilitation graded care works through multiple mechanisms. Exercise training, a core component of cardiac rehabilitation, has been shown to activate anti-inflammatory pathways. Exercise increases circulation, facilitates the removal of inflammatory mediators, and improves endogenous antioxidant capacity, thereby decreasing levels of inflammatory markers such as CRP and TNF-α [24]. In addition, regular exercise further reduces the chronic inflammatory state by promoting vascular endothelial function, improving insulin resistance, and promoting lipid metabolism [25]. At the molecular level, cardiac rehabilitation graded care may reduce the expression of inflammatory factors by affecting cell signaling pathways. For example, exercise reduces the activity of NF-κB (nuclear factor κB), a key transcription factor that promotes the expression of several inflammatory cytokines, including TNF-α and CRP.

CRP and TNF-α are important inflammatory factors and markers of myocardial injury. Their elevated levels can damage cardiomyocytes and inhibit myocardial contractility, resulting in decreased cardiac function [26]. Cardiac hypofunction directly affects the body’s exercise tolerance [27]. Inflammatory reactions lead to the production of a large number of muscle injury-related factors, CK (creatine kinase) and LDH (lactate dehydrogenase), in the body, which can also damage skeletal muscles. Muscle hypofunction will also directly lead to decreased exercise endurance [28]. TNF-α can inhibit skeletal muscle proliferation and promote protein degradation [29]. This results in reduced muscle mass and decreased muscle strength, which affect exercise tolerance [30]. Therefore, it is speculated that stratified cardiac rehabilitation can improve exercise endurance by reducing the level of inflammatory factors, protecting myocardial and skeletal muscle cells, and improving microcirculation and oxygenation function [31]. Different exercise tolerance indexes reflect different aspects of aerobic exercise ability, but they are closely related to cardiopulmonary function and muscle status. It can be seen that reducing inflammation is an important way to improve exercise tolerance, and there is a complex biological internal relationship between the two. At the same time, this study further observed the improvement in ED and VO_2max_ in observation group patients, but the placebo effect could not be completely excluded due to study restrictions. Considering that psychological expectations of patients may also have an impact on their physiological status, the improvement in exercise tolerance may still be affected by the placebo effect of patients’ own psychology and diet. But this study considered the effect of placebo effects in the statistical analysis and used appropriate methods to adjust the α levels to control the risk of multiple comparisons and type I errors. This will help us to more accurately assess the differences between the experimental and control groups and determine how much the observed effects can be attributed to the placebo effect.

Compared with the study of Malandish et al. [32], the results of this study only focused on the elderly population with CHD and used not only exercise intervention but also more specific and comprehensive intervention population and intervention measures. At the same time, the observation indicators of this study cover CRP, TNF-α, and a number of exercise tolerance indicators, with a wider coverage. Compared with the results of Bhati et al. [33], this study showed that stratified cardiac rehabilitation had a more significant effect on TNF-α, which may be related to the fact that only elderly patients were included in this study. At the same time, this study found that resistance exercise had anti-inflammatory effects and reduced the degree of cardiac autonomic dysfunction [34]. This study confirms that stratified cardiac rehabilitation can not only reduce inflammatory factors but also significantly improve the exercise tolerance of elderly patients. This finding enriches the application scope of cardiac rehabilitation care. The exercise endurance of the control group also improved, which was related to the drug treatment, lifestyle, and exercise training, but the effect was insufficient compared with the observation group. This research demonstrates that stratified cardiac rehabilitation enhances the prognosis for elderly patients with CHD, supporting the broader adoption of this rehabilitation model. The findings also offer insights into potential molecular mechanisms underlying cardiac rehabilitation, aiding future studies in detailing its effects.

Nevertheless, the limited sample size of this study, consisting of only 94 participants, raises concerns about the representativeness of the results. Future research could utilize a multi-center approach to expand the sample size and enhance the robustness of the findings. This randomized controlled trial indicates that stratified cardiac rehabilitation effectively lowers the CRP and TNF-α levels and boosts exercise tolerance in elderly patients with CHD, suggesting it contributes significantly to these improvements. These outcomes confirm the causal link between stratified cardiac rehabilitation and reductions in inflammatory markers as well as increased exercise tolerance. Further investigations could reinforce this causal connection by enlarging the participant pool and extending the duration of follow-up. But this study considered the effect of placebo effects in the statistical analysis and used appropriate methods to adjust the α levels to control the risk of multiple comparisons and type I errors. This will help us to more accurately assess the differences between the experimental and control groups and determine how much of the observed effects can be attributed to the placebo effect.

In this study, only short-term follow-up was performed, and the long-term effect needs further observation. It is suggested to set up a longer follow-up period to observe the long-term effect of graded cardiac rehabilitation care. Additionally, this study concentrated solely on four inflammatory markers, CRP, IL-6, fib, and TNF-α, omitting a broader analysis of the inflammatory profile. Future research should include a wider array of inflammatory markers. The current study also involved only a short-term follow-up; assessing the long-term impacts of stratified cardiac rehabilitation is necessary. A longer follow-up period is recommended for observing the sustained effects of rehabilitation. In addition, the study involved only 94 patients, which limits its statistical power and generalizability. A multicenter trial with a larger, more diverse cohort will improve the applicability of the findings to a wider population. The sample size still needs to be expanded in the future to avoid potential bias in the results.

## 5 Conclusions

The application of cardiac rehabilitation graded care in elderly patients with CHD not only significantly improves the levels of inflammatory markers and exercise tolerance but also improves the quality of survival of patients, which is an effective nursing intervention. Future studies can further explore the application effect of graded cardiac rehabilitation care in different patient groups, as well as its mechanism of action at the cellular and molecular levels, to provide more scientific basis for clinical practice. At the same time, it also provides strong evidence to support the clinical implementation of cardiac rehabilitation and suggests that cardiac rehabilitation graded care should be widely used in the treatment and rehabilitation of patients with CAD.
